# Nitrate Toxicity in Ruminant Production Systems

**DOI:** 10.3390/ani16152306

**Published:** 2026-07-26

**Authors:** Gavin Haines, Timothy DelCurto, Sam Wyffels, Megan Van Emon

**Affiliations:** Department of Animal and Range Sciences, Montana State University, Bozeman, MT 59717, USA; timothy.delcurto@montana.edu (T.D.); sam.wyffels@montana.edu (S.W.)

**Keywords:** nitrate accumulation, nitrate toxicity, ruminants

## Abstract

Nitrate toxicity remains a significant concern in ruminant livestock production, especially under conditions of high nitrogen fertilization or environmental stress that promote nitrate accumulation in forage. This review explores the physiological, environmental, and management factors influencing nitrate uptake in plants and its conversion in the rumen. The authors highlight variability in plant species, forage types, and feeding systems, along with inconsistencies in toxicity thresholds and management recommendations. This review examined differences in species susceptibility, ruminal microbial efficiency, and the need for practical, evidence-based strategies. Finally, this review emphasizes the need for continued research to develop consistent guidelines and improve nitrate management in modern cattle systems.

## Simple Summary

Nitrate accumulation in forages under stress conditions such as drought, frost, or heavy fertilization poses serious health risks to livestock, particularly cattle. Ingested nitrate can impair oxygen transport in the blood, leading to reduced performance, reproductive losses, or even death. Even though the economic cost of nitrate toxicity is well known, there is limited research-based information on the effects of nitrate toxicity and management of high nitrate feeds in livestock production. This review outlines the factors influencing nitrate buildup and toxicity, while emphasizing the need for clear, evidence-based guidelines to support safe livestock production.

## 1. Introduction

Nitrate toxicity presents a serious threat to ruminant health, especially in cattle consuming forages grown under conditions of high nitrogen fertilization and/or environmental stress. When nitrate is not effectively reduced to ammonia in the rumen, it can accumulate as nitrite and be absorbed in the bloodstream, leading to methemoglobinemia, a condition that hinders oxygen transport and can result in decreased productivity, reproductive failure, or death [1]. Initial research on nitrate toxicity was conducted by Bradley and colleagues in 1940, and much of the subsequent understanding has been built on these early studies [2]. However, a lack of sustained research since the 1970s has contributed to inconsistencies and gaps in current feeding recommendations.

Recommended nitrate intake levels for cattle vary widely, largely because many published recommendations provide limited information regarding the research and experimental conditions used to establish tolerance thresholds. For example, while some researchers suggest a maximum of 10 g of NO_3_^−^ per 100 kg of body weight per day, others have reported that cattle can tolerate up to 90 g per day under certain conditions [3]. This inconsistency is also seen in state-level guidelines in the USA; for instance, Montana recommends a maximum of 10,000 mg/kg of nitrate in feed for non-pregnant cattle, whereas neighboring Wyoming recommends up to 17,720 mg/kg [4,5]. Despite similarities in geography and cattle production systems, these distinct thresholds can confuse producers. Such variation highlights the influence of different research methods, environmental factors, and assumptions about animal adaptability. It also emphasizes the need for clearer, more standardized, and context-specific recommendations to guide safe nitrate management in livestock systems.

**Figure 1:**
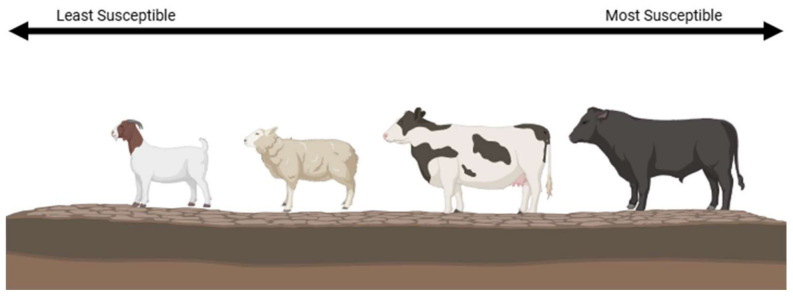
This image demonstrates the susceptibility of ruminants to nitrate toxicity.

Nitrate toxicity affects a variety of livestock species, though susceptibility differs across animals (Figure 1). Ruminants are particularly vulnerable because microbial fermentation in the rumen converts nitrate into nitrite, which can enter the bloodstream and disrupt oxygen transport [6]. Among ruminants, beef cattle and dairy cattle are especially susceptible due to their large rumen capacity and bulk-grazing behavior, which allows them to consume large quantities of forage rapidly [7]. Beef cattle may be at greater risk of nitrate toxicity because extensive grazing systems often expose animals to sudden increases in forage nitrate concentrations, providing less opportunity for rumen microbial adaptation [7]. Dairy cattle are also at risk; however, their diets typically include high-energy feeds that may enhance the ability of rumen microbes to metabolize nitrate more efficiently [8]. Sheep, on the other hand, are less susceptible because they are selective grazers that consume primarily leaf material and smaller, more frequent meals, reducing the likelihood of acute nitrate overload [7]. Compared with cattle and sheep, goats are less likely to develop nitrate poisoning under grazing conditions because their selective browsing behavior reduces the probability of ingesting large quantities of high-nitrate plants [9]. Because there is reduced risk with dairy cattle, sheep, and goats, there is little to no research relative to nitrate toxicity or feeding recommendations in these species. Therefore, the majority focus on beef cattle production systems.

Nitrate toxicity remains a significant concern in livestock production systems, particularly for ruminants. As forage management practices evolve and environmental pressures increase, understanding the factors that contribute to nitrate accumulation and its impact on animal health is critical. This literature review examines the biological processes involved in nitrate accumulation in plants, the mechanisms of toxicity in ruminants, and the practical strategies used to manage and reduce the risk of nitrate poisoning.

## 2. Methods

A narrative literature review was conducted using Google Scholar as the primary database. Searches were performed using the keywords “nitrate toxicity,” “nitrate accumulation,” and “ruminants,” in various combinations with Boolean operators (AND, OR), including phrases such as “nitrate toxicity AND cattle,” “nitrate accumulation AND forage,” and “ruminant nitrate metabolism.” Articles published in English between 1940 and 2025 were considered. Titles and abstracts were screened for relevance, and full texts were reviewed when studies addressed nitrate concentrations in feed, nitrate metabolism, or toxicological effects in ruminant species (cattle, sheep, or goats). Studies were excluded if they focused on non-ruminant species, human health, or did not present original data or relevant reviews on nitrate toxicity. From each included study, information was extracted on ruminant species, forage or feed type, nitrate concentration, exposure conditions, and reported health or production outcomes. The results were synthesized qualitatively by grouping studies according to forage type, nitrate levels, and reported toxicological responses to identify common patterns and thresholds associated with nitrate toxicity and accumulation in ruminants.

## 3. Nitrate Accumulation in Plants

**Figure 2:**
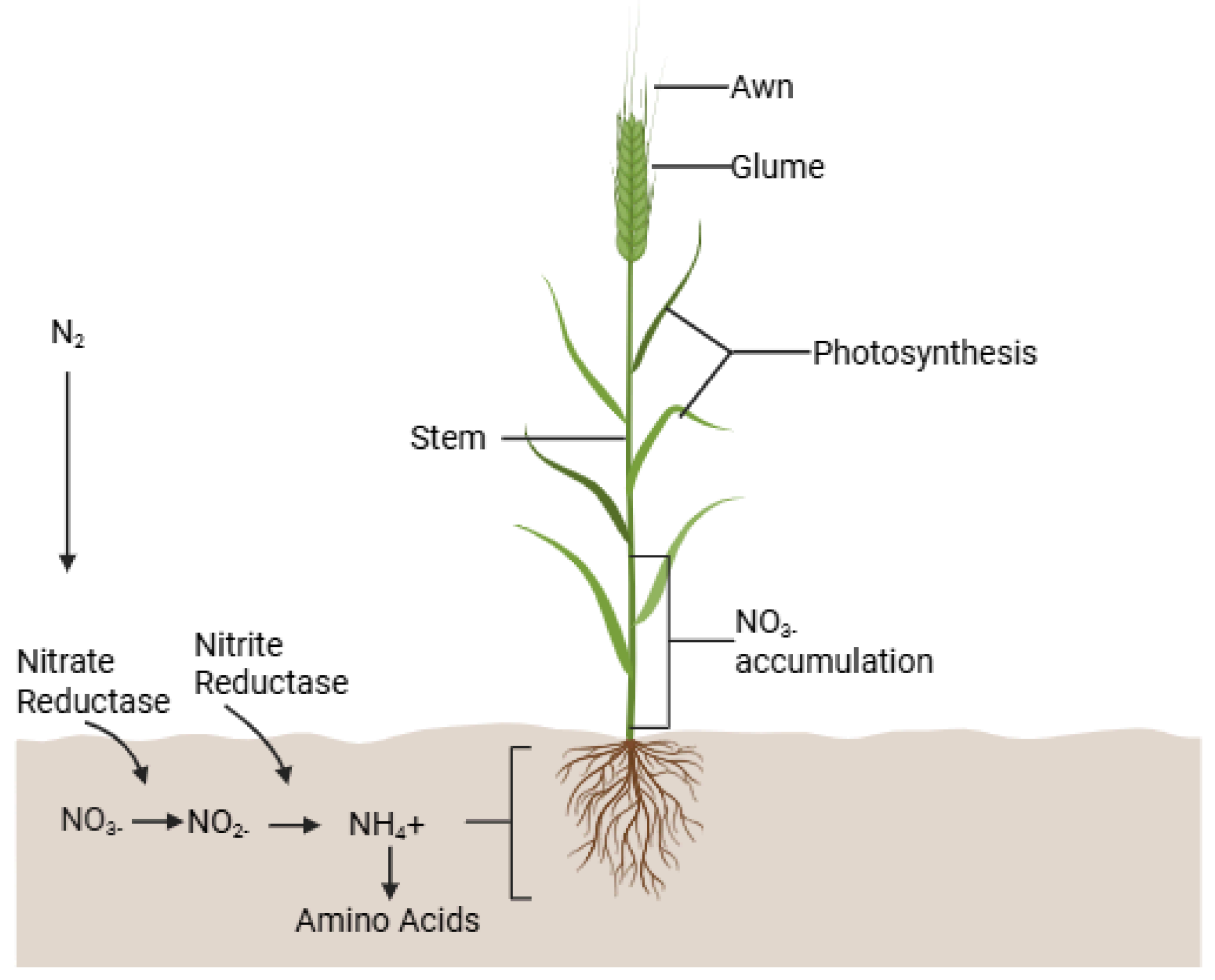
The process of nitrogen assimilation into and out of the soil and, in turn, plant uptake of nitrogen from the soil. The primary location of the plant where nitrates accumulate in the lower stem, when environmental conditions favor nitrate accumulation [10].

Every plant has the potential to accumulate nitrate, and the process will start with the plant roots extracting nitrate from the soil (Figure 2). Then nitrate assimilation will occur by the enzyme nitrate reductase (NR), converting nitrate to nitrite. Nitrite will then be moved into the stroma of the chloroplast and reduced to ammonia by the enzyme nitrite reductase (NIR). In turn, NIR reduces nitrate to nitrite by using electrons from NAD(P)H, and the reduced ferredoxin will be generated due to photosynthetic electron transport. Photosynthetic electron transport is the electron donor for nitrite reduction. Ammonia will be combined with glutamate to be assimilated into glutamine [10]. It is important to consider that the process of nitrate accumulation is common for all graminoid species, and the types of plants and environments that elevate nitrates are predictable.

## 4. Factors Impacting Nitrate Accumulation

Nitrate accumulation in plants is influenced by a variety of factors, many of which are directly linked to stressful growing conditions. Key environmental variables include light intensity, water availability, temperature, and soil properties [11]. Specific stressors that can trigger nitrate buildup include shading, drought, frost, extended periods of unusually cold temperatures, physical damage to the plant, and imbalanced soil nutrients [4]. Further discussion on plant physiological stressors follows.

### 4.1. Light Intensity

The conversion of nitrates to amino acids depends on the process of photosynthesis. When this process is disrupted at night, or if there is a lack of sunlight during the day, plants will continue to accumulate nitrates but cannot be converted to amino acids. In barley seedlings, nitrate accumulation increased 2.3 times in the dark; however, in the light, 80% of accumulated nitrates were reduced [12]. Nitrate levels are greatest in the morning before sunrise, with accumulation occurring in the bottom one-third of the stem [13]. Therefore, nitrate-accumulating plants may pose a greater risk for potential nitrate toxicity to ruminants when grazed early in the day or harvested early in the day. However, a study on wheat root uptake suggests that net nitrate root uptake increased in daylight hours and decreased in darkness [14]. Therefore, the physiological response of above-ground biomass versus root biomass warrants further investigation.

### 4.2. Temperature

Temperature plays a significant role in nitrate accumulation by influencing both nitrate uptake and its conversion within the plant. Optimal temperatures for warm-season plants are 21–32 °C, and cool-season plants are 21–24 °C [15]. Optimal temperatures support active photosynthesis and the enzymatic processes of reducing nitrate into plant proteins. However, under cold stress or prolonged low temperatures, nitrate reductase activity slows, reducing the plant’s ability to assimilate nitrate efficiently [11]. Heat stress has been shown to negatively impact plant growth and nutrient dynamics by reducing photosynthesis, root development, and nutrient uptake efficiency. These effects are often associated with decreased function and abundance of nutrient-uptake proteins and nitrogen-assimilating enzymes, ultimately reducing nutrient availability and overall crop quality [16]. As a result, nitrate concentrations may build up in plant tissues, especially in the lower stems and leaves. Similarly, sudden temperature drops, such as frost events, can damage plant tissues and further inhibit nitrate metabolism. Therefore, when forage crops experience abnormal or fluctuating temperatures, there is an increased risk of elevated nitrate concentrations, which should be carefully monitored to prevent toxicity in livestock.

### 4.3. Water

Water scarcity, particularly during drought, places considerable stress on plants and can interfere with nutrient uptake and essential metabolic processes. Since water may comprise 80–90% of a plant’s total weight, it plays a critical role in nearly all physiological functions [11]. Short-term drought conditions often lead to nitrate accumulation in plant tissues because roots may still absorb nitrate from the soil, but limited water availability restricts photosynthesis, the process necessary for converting nitrate into proteins [17]. In contrast, extended drought reduces both nitrate uptake and assimilation, as water is essential not only for photosynthesis but also for the mass flow and diffusion mechanisms that move nitrate from the roots [18]. Over time, as water deficiency persists, the plant’s ability to accumulate and process nitrate declines.

### 4.4. Soil Nutrient Availability

Soil nutrient availability plays a significant role in the accumulation of nitrates in plants, particularly through the practice of over-fertilization. Excessive application of nitrogen fertilizers can also lead to increased nitrate uptake by plants, surpassing their capacity for nitrate reduction and assimilation, which results in nitrate accumulation in plant tissues [11]. However, over-fertilization is not the only contributor; the type of nitrogen fertilizer used also influences nitrate accumulation [19]. For instance, nitrate-based fertilizers tend to increase nitrate content in plants, while ammonia-based nitrogen fertilizers have been shown to reduce nitrate accumulation [20]. Therefore, the type of fertilizer should be considered when applying it to plants that are potential nitrate accumulators, as this may significantly impact the potential for nitrate toxicity.

## 5. Plant Factors Impacting Nitrate Accumulation

Several plant-related factors influence the potential for nitrate accumulation in forages, affecting their safety and nutritional value for livestock. Characteristics such as stage of maturity, photosynthetic pathway (C3 vs. C4), plant life cycles (annual vs. perennial), and species-specific traits all play a role in how efficiently plants take up and assimilate nitrate. Understanding these differences is essential for identifying high-risk forages and implementing proper management practices to prevent nitrate toxicity in livestock.

### 5.1. Plant Maturity

Photosynthesis in the leaves plays a critical role in converting nitrates into amino acids (Figure 1), and a plant’s age and leaf development influence this process. One key factor affecting nitrate assimilation is the distribution of dry matter within the plant. During the early stages of growth, dry matter is primarily allocated to roots and stems, as the plant focuses its assimilation efforts on root and stubble development [21]. As the plant matures, this distribution gradually shifts, leading to more dry matter accumulating in the leaves. Since photosynthesis occurs in the leaves, greater leaf development enhances the plant’s ability to convert nitrate into organic forms. Therefore, as leaf mass increases with age, nitrate assimilation increases compared to the early growth stages when biomass is concentrated in non-photosynthetic tissues [21]. This relationship between plant maturity and nitrate metabolism is crucial for timing forage harvests to minimize the risk of nitrate toxicity. Clearly additional research is needed with respect to plant species, phenological age, and environmental stress interactions.

### 5.2. Cool- and Warm-Season Species

Every plant has the potential to accumulate high levels of nitrate; however, the efficiency with which different species convert nitrate into plant protein varies. Warm-season, C4 plants such as maize are more efficient at this conversion than cool-season, C3 plants like oats. This is largely due to their enhanced capacity for CO_2_ fixation and water use efficiency. However, C4 plants are more tolerant to environmental stressors, such as cloudy days, thermo stress, and precipitation, which can pose a toxic risk to grazing livestock [22]. For example, C4 species such as maize and sorghum commonly present nitrate risk under fertilizer/drought scenarios.

### 5.3. Plant Life Cycles

Annual and perennial plant species differ significantly in their growth rate, nutrient uptake, and physiological characteristics, all of which influence their potential for nitrate accumulation and forage value. Annuals are more prone to accumulating high nitrate levels due to their rapid growth and greater nutrient demand. Garnier and coworkers [23] found that annual grasses not only grew faster but also had greater nitrate absorption rates than their perennial counterparts. While both annual and perennial forages can accumulate nitrates under certain conditions, the accelerated uptake in annuals makes them especially susceptible, particularly during periods of environmental stress. Therefore, annual forages should be monitored closely and routinely tested for nitrate concentrations before being fed to livestock to minimize the risk of toxicity.

### 5.4. Species-Specific Traits

Annual plants in cropping systems are at the greatest risk of accumulating high nitrate levels. This is due to fertilization, combined with environmental and physiological factors that influence nitrate uptake and conversion. Warm-season annual crops such as maize, sudangrass, soybeans, and sorghum, and cool-season annual crops such as barley, wheat, rye, and flax are particularly prone to nitrate accumulation [13]. While some differences among these species relate to their photosynthetic pathways, another key distinction lies in how they distribute dry matter. As plants mature, the distribution of dry matter becomes a critical factor in nitrate conversion. Species-specific differences in nitrate accumulation can often be explained by these patterns, particularly because morphological variation in nitrate metabolism is closely tied to leaf yield [24].

Planting cover crops is a common management practice in cropping systems, valued for its dual benefits of enhancing soil health and providing forage for livestock. However, a study conducted by Wyffels et al. [25] found that both cool-season and warm-season cover crop mixtures can accumulate nitrate at high risk levels. Elevated nitrate concentrations were especially apparent under unfavorable weather conditions, such as drought and below-optimal temperatures, particularly affecting both C3 and C4 plant species. Since cover crops are typically grown on fertilized fields, conditions that favor growth may also increase the risk of nitrate accumulation. Therefore, producers should exercise caution and test nitrate levels before harvesting cover crops or allowing livestock to graze.

## 6. Nitrate Conversion in Rumen

**Figure 3:**
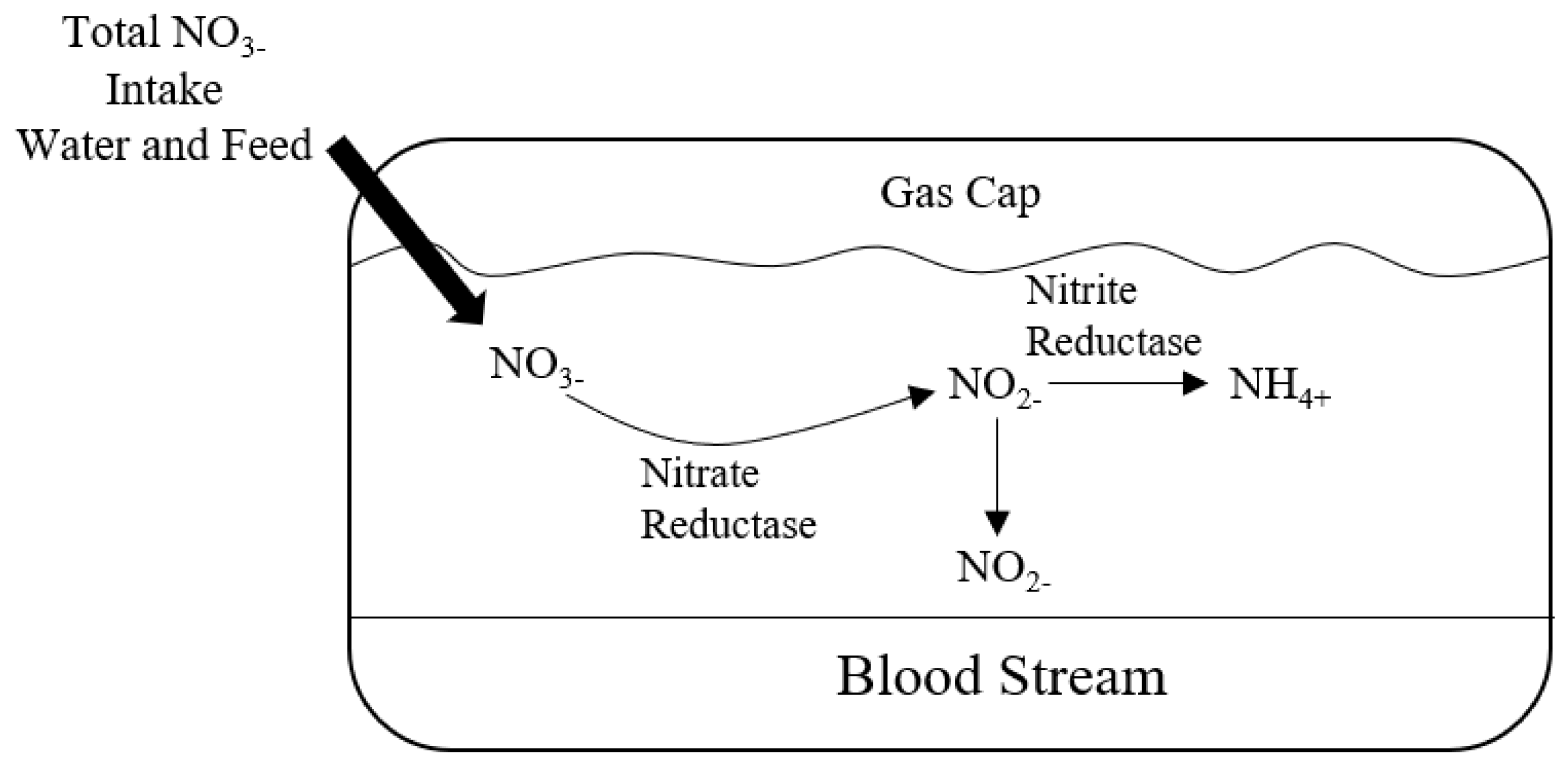
This image depicts the pathway of nitrate conversion within the rumen, highlighting how elevated levels of nitrite can be absorbed through the rumen wall into the bloodstream, contributing to nitrate toxicity [26].

Although nitrate, nitrite, and ammonia can all cause toxicity problems for ruminant animals, nitrite is the most problematic form of non-protein nitrogen. Nitrate toxicity occurs when an animal consumes feed high in nitrates in a short amount of time. In the rumen, nitrate is converted to nitrite, then to ammonia (Figure 3 [26]). The stage of conversion that may cause toxicity to the animal is the rate-limiting step of nitrite to ammonia. Nitrate toxicity occurs when nitrite accumulates in the rumen and is absorbed into the bloodstream and attaches to hemoglobin [26]. When nitrite binds with hemoglobin, hemoglobin is converted to methemoglobin, which is unable to carry oxygen. However, while high levels of nitrate can pose a serious risk of toxicity, low levels represent minimal risk (5000 mg/kg [4]) to animal health.

## 7. Nitrate Toxicity in Water

Nitrate toxicity from water is generally low risk when cattle drink from well water, but the danger increases with surface water sources, particularly those influenced by runoff from fertilized fields, feedlots, manure lagoons, or silos. Water containing less than 100 ppm of nitrate–nitrogen (NO_3_-N) is typically considered low risk for livestock [27]. While water is not the most common source of nitrate toxicity, producers should still take preventive measures by testing water sources and ensuring cattle are not simultaneously consuming high-nitrate feed [27].

## 8. Nitrate Toxicity and Symptoms in Ruminants

### 8.1. Conversion of Hemoglobin to Methemoglobin

When nitrite binds to hemoglobin, it converts hemoglobin into methemoglobin, a form that cannot transport oxygen. As methemoglobin increases in the blood to 40–60% of hemoglobin, clinical signs of nitrate toxicity may be observed [26]. Death may occur in cattle when the percentage of hemoglobin converted to methemoglobin is 70–90% [26]. Therefore, increased methemoglobin concentrations may be the primary cause of nitrate toxicity and not the increased nitrite concentrations within the rumen. However, with the difficulty measuring methemoglobin in real time, it is challenging to ascertain when symptoms or death may occur.

### 8.2. Symptoms

When the ratio of methemoglobin to hemoglobin reaches threshold levels, producers may begin to observe clinical signs of nitrate toxicity. Early symptoms may result in watery eyes, reduced appetite, reduced milk production, rough hair coat, weight loss or no weight gain, night blindness, or abortion [4]. Because most of the hemoglobin can no longer carry oxygen to the body tissues and organs, symptoms common to hypoxia arise. Acute symptoms, including accelerated pulse rate, difficulty breathing, shortness of breath, muscle tremors, weakness, staggering gait, cyanosis, and death, are common with nitrate toxicity [4]. Although the impact of nitrate toxicosis is a challenge for the livestock industry, strategies to minimize the occurrence of nitrate and treatments to prevent toxicosis do exist.

### 8.3. Nitrate Toxicity in Pregnant Cattle

Nitrate toxicity presents a heightened risk for pregnant cattle due to their increased physiological sensitivity during gestation. The hypoxic stress caused by nitrate-induced methemoglobinemia may impair fetal development and significantly raise the likelihood of abortion [28]. As was illustrated in Table 1, recommended maximum tolerable nitrate levels for pregnant cattle are considerably lower than those for non-pregnant animals, ranging from 5000 mg/kg to 15,505 mg/kg of feed. In addition to fetal loss, nitrate toxicity potentially negatively impacts conception rates, reducing reproductive efficiency [28]. Fetal loss may occur due to the lack of oxygen transport to the reproductive tract to support pregnancy. These risks highlight the critical need to monitor nitrate levels in forage and water, particularly during drought conditions or other environmental stressors, to safeguard both maternal health and reproductive success.

### 8.4. Nitrate Toxicity in Small Ruminants

Although sheep and goats are susceptible to nitrate toxicity, poisoning occurs less frequently than in cattle because of their selective feeding behavior rather than physiological resistance. Sheep consume smaller, more frequent meals, while goats preferentially browse leaves and shrubs, reducing the likelihood of ingesting large amounts of nitrate-rich forage [7,9]. Nevertheless, both species remain susceptible when excessive nitrate is consumed, with toxicity resulting from nitrite accumulation, methemoglobin formation, and impaired oxygen transport. Experimental studies in goats have demonstrated that both acute and chronic nitrate exposure cause pathological changes, including pulmonary edema, hepatic and renal degeneration, widespread congestion, and methemoglobinemia, indicating that prolonged exposure to sublethal nitrate concentrations can impair organ function [32,33]. Despite these findings, research on nitrate toxicity in small ruminants remains limited, and most feeding recommendations continue to be extrapolated from cattle studies.

### 8.5. Dairy Cattle

Dairy cattle are susceptible to nitrate toxicity through the same mechanism as beef cattle, in which ruminal nitrate is reduced to nitrite and, under normal conditions, to ammonia. A balanced diet is one that meets the animal’s nutrient requirements while supplying sufficient fermentable energy to support ruminal microbial activity and efficient nitrate-to-ammonia reduction. Because dairy diets typically contain greater amounts of fermentable carbohydrates, they may better support microbial nitrate reduction and reduce the risk of nitrite accumulation. Dietary management is essential for minimizing nitrate toxicity while maintaining production. Sharifi et al. [34] reported that dietary nitrate supplementation did not reduce milk yield and reduced methane production by serving as an alternative hydrogen sink when incorporated into balanced dairy rations. In contrast, Davison et al. [28] found that chronic high nitrate intake reduced conception rates and increased abortions and mortality in dairy heifers, although milk production and growth were largely unaffected. These studies demonstrate that nitrate toxicity in dairy cattle depends not only on nitrate concentration but also on diet composition and management.

### 8.6. Meat Quality

Research evaluating the direct effects of nitrate toxicity on meat quality remains limited. Most studies have focused on animal health, methane mitigation, and production performance rather than carcass characteristics. Lee and Beauchemin [35] reported that dietary nitrate supplementation generally had no adverse effects on dry matter intake or live weight gain when animals were gradually adapted to nitrate-containing diets, indicating that production performance can be maintained under proper management. Furthermore, although nitrate supplementation reduced enteric methane emissions, the energy conserved from reduced methane production did not translate into measurable improvements in animal growth or carcass production. Current evidence does not demonstrate consistent effects of dietary nitrate on carcass characteristics or meat quality traits such as marbling, tenderness, color, or sensory attributes. Therefore, additional research is needed to determine whether long-term nitrate supplementation or chronic nitrate exposure influences carcass composition and meat quality in ruminants.

## 9. Treatment for Nitrate Toxicity in Cattle

Nitrate toxicity may be treated by administering methylene blue. A study found that administering varying doses of methylene blue between 4–22 mg/kg body weight, depending on the severity of the toxicity, was able to treat acute signs of nitrate toxicity [36]. Researchers observed that nitrite and methemoglobin concentrations were reduced within 12 h after the dosage administration [36]. Therefore, methylene blue may be used as a potential treatment for nitrate toxicity. In addition, oral gavage flushes of cold water reduce microbial activity, which in turn reduces the conversion of nitrate to nitrite [36]. Both potential treatments may have some success; however, for grazing livestock, treatment may be challenging due to the lack of facilities or the difficulty in observing symptoms during or after feeding nitrate-containing feeds. However, treatment for nitrate toxicity should be conducted with a veterinarian consultation.

## 10. Differing Feeding Recommendations

### 10.1. USA Feeding Recommendations

The variability in maximum tolerable nitrate concentrations for cattle across different classes of livestock and regions of North America highlights the complexity of managing nitrate toxicity, particularly when considering animal physiological status. As shown in Table 1, recommended limits for non-pregnant cattle range from 10,000 mg/kg of feed in Montana and Oklahoma to as high as 17,720 mg/kg of feed in Wyoming, North Dakota, and South Dakota. For pregnant cattle, the thresholds are consistently lower, ranging from 5000 mg/kg to 15,505 mg/kg of feed. This can be from the increased susceptibility of gestating animals to nitrate toxicity and the associated risk of abortion.

### 10.2. International Feeding Recommendations

International recommendations for managing nitrate-containing feeds vary among countries but consistently emphasize gradual adaptation and sound feeding management to minimize the risk of toxicity. In Canada, the Saskatchewan Ministry of Agriculture [37] considers forage containing less than 5000 mg NO_3_/kg dry matter (DM) to be generally safe for cattle, while forage containing 5000 to 10,000 mg NO_3_/kg DM should be fed cautiously, and forage exceeding 10,000 mg NO_3_/kg DM requires dilution with low-nitrate feeds, gradual adaptation over 1 to 2 weeks, and adequate dietary energy to promote microbial reduction in nitrate to ammonia. In Australia, recommendations from Meat & Livestock Australia [38] focus on the safe use of supplemental nitrate rather than naturally occurring nitrate in forage. These guidelines recommend gradually adapting cattle over approximately 14 days, limiting nitrate supplementation according to body weight and forage digestibility, providing adequate sulfur to support rumen microbial activity, and avoiding supplementation to hungry or stressed cattle. In contrast, the European Food Safety Authority (EFSA) [39] does not establish maximum allowable nitrate concentrations in feed but instead uses a science-based risk assessment approach. EFSA identified a benchmark dose lower confidence limit (BMDL_10_) of 64 mg nitrate/kg body weight/day for cattle based on methemoglobin formation and concluded that fixed nitrate thresholds for feed cannot be established because toxicity is influenced by factors such as forage type, diet composition, rumen microbial adaptation, dietary energy, and overall feeding management. Despite differences in approach, international recommendations consistently emphasize forage testing, gradual adaptation, adequate dietary energy, and proper management practices as the primary strategies for reducing the risk of nitrate toxicity in cattle.

## 11. Feed Conservation Methods and Diet

### 11.1. Non-Dried Forage vs. Dried Harvested Forage

Forage type, non-dried forages vs. dried harvested forages, may have an impact on the release rate of nitrate into the rumen. Previous work suggests non-dried forage released 10–30% of accumulated nitrates in 20 min in distilled water, while dried hay released 80% of nitrates in 20 min [40]. This indicates that cattle consuming dried forage are at a greater risk of nitrate toxicity than cattle grazing fresh forage. This may, in part, be due to the rehydration of forages as they are consumed, releasing nitrates within the rumen. With this rapid influx of nitrates into the rumen, enhancing microbial efficiency within the rumen may be a possible way to reduce the potential for nitrate toxicity.

### 11.2. Concentrate Diet

Enhancing microbial efficiency in the rumen may help reduce the risk of nitrate toxicity. Therefore, changing the diet could increase the rate at which nitrite is converted to ammonia. Concentrates like grain sources can help minimize the buildup of methemoglobin in the blood by speeding up the conversion of nitrite to ammonia. Burrows and colleagues [26] observed a linear decrease in both ruminal nitrate and methemoglobin as corn supplementation increased in cattle. Additionally, researchers have noted that adding diets rich in electron donors such as formate, lactate, and hydrogen can improve the reduction in nitrite to ammonia and lower exposure to toxic nitrite levels [41]. Thus, one way to reduce nitrate toxicity is to include high-energy feeds when feeding forages containing nitrates.

## 12. Ruminal Microbiome

### 12.1. Nitrate- and Nitrite-Reducing Microbes

The microbial community within the rumen plays a crucial role in converting nitrates to ammonia, which is essential for lowering the risk of nitrate toxicity in ruminants. Key nitrate-reducing bacteria include *Selenomonas ruminantium*, *Veillonella parvula*, and *Wolinella succinogenes* [42]. As the ruminal microbes play a key role in the reduction in nitrate and nitrite, factors that may affect the efficiency of the microbial population are the availability of energy and ruminal pH.

### 12.2. Electron Donors

The efficiency of this conversion relies heavily on the availability of electron-donating substrates, which supply the necessary reducing power for these biochemical reactions. Moreover, the overall physiological capacity of the microbial population affects the success of these reduction processes [43]. Common electron donors that support nitrate and nitrite reduction include fermentable compounds such as formate, lactate, and hydrogen [43]. Research by Iwamoto et al. [44] identified formate and hydrogen as the most effective electron donors, especially in enhancing nitrite reduction. Therefore, it is necessary to consider both microbial health and dietary composition when managing nitrate-rich diets, as optimizing electron donor availability can play a critical role in minimizing the risk of nitrate toxicity in ruminants.

### 12.3. Methane Mitigation

Dietary nitrate has gained attention as a nutritional strategy to reduce enteric methane emissions in ruminants because it functions as an alternative hydrogen sink during ruminal fermentation. Rather than hydrogen being utilized by methanogenic microorganisms to produce methane, hydrogen is instead used to reduce nitrate to nitrite and ultimately to ammonia [35]. This process can substantially reduce methane production while simultaneously providing ammonia for microbial protein synthesis. Olijhoek et al. [45] demonstrated that increasing dietary nitrate reduced enteric methane emissions by approximately 30% while having minimal effects on nutrient digestibility and ruminal fermentation when dairy cattle gradually adapted to nitrate-containing diets. However, because nitrite reduction is the rate-limiting step in nitrate metabolism, improper adaptation or excessive nitrate intake may result in nitrite accumulation and an increased risk of methemoglobinemia. Therefore, successful implementation of nitrate as a methane mitigation strategy depends on gradual adaptation, adequate fermentable energy, and careful dietary management to maximize methane reduction while minimizing the risk of nitrate toxicity.

### 12.4. pH

Ruminal pH plays a crucial role in regulating the microbial reduction in nitrate and nitrite. Iwamoto et al. [44] found that the reduction in both compounds was most efficient at a neutral ruminal pH of 7.0, with activity declining significantly as ruminal pH became more acidic. This reduced efficiency is likely due to the inhibitory effects of low pH on microbial growth and fermentation, which in turn limit the production of essential electron donors such as formate, lactate, and hydrogen. When ruminal pH falls below 6.0, these fermentation pathways are suppressed, diminishing both the population of nitrate-reducing microbes and the reducing power needed for nitrate and nitrite conversion. However, caution is warranted when feeding high-energy diets, as they can have dual effects on nitrate metabolism, providing electron donors that support reduction processes, but also potentially lowering rumen pH and impairing microbial function if not properly balanced.

### 12.5. Adaptation to High-Nitrate Diets

When ruminants are adapted to high-nitrate diets, the adaptation occurs primarily within the rumen microbial population, rather than in the animal’s own metabolism. With gradual exposure to increasing nitrate levels, the rumen microbial community shifts to favor nitrate- and nitrite-reducing species such as *Selenomonas ruminantium*, *Veillonella parvula*, and *Wolinella succinogenes*, which are highly efficient at converting nitrite into ammonia. As these microbes increase in abundance and activity, harmful nitrite accumulation is prevented, allowing nitrate to be safely used as a nitrogen source. In a study by Alaboudi and Jones [46], sheep were acclimated to 2.5 g KNO_3_/kg body weight by increasing dietary nitrate by 0.5 g KNO_3_/kg body weight every two weeks. Rumen fluid from adapted sheep reduced nitrate three times faster and nitrite five times faster than rumen fluid from non-adapted sheep. These results demonstrate that the animal tissues do not significantly change their capacity to reduce nitrate; rather, protection comes from the rumen microbial community becoming more efficient at handling nitrate.

## 13. Managing Nitrate Concentrations

If environmental stress or heavy fertilization raises concerns about high nitrate levels in forage, several management strategies can help reduce the risk of toxicity:

- Test both soil and forage to determine nitrate concentrations before harvest, feeding, or grazing.
- Delay harvest to allow for further plant maturity and nitrate metabolism, as older plants typically contain lower nitrate levels.
- Avoid cutting the lower portions of the stalk, where nitrate tends to concentrate most heavily.
- Consider ensiling the forage, as the fermentation process can help reduce nitrate content before feeding.

While these management strategies can help reduce nitrate levels and the risk of toxicity, they have limitations. Testing may not always be timely or accessible. Delaying harvest can reduce forage quality, and avoiding lower stalks may lead to yield loss. Additionally, ensiling is not always effective or feasible, depending on resources and weather conditions.

## 14. Economic Impacts of Nitrate Toxicity

Nitrate toxicity can have significant economic consequences for livestock producers by reducing animal health, productivity, and reproductive performance. Acute poisoning may result in sudden death, while chronic exposure to elevated nitrate concentrations can reduce feed intake, impair weight gain, increase abortion rates, and decrease overall herd performance [47]. These production losses are compounded by veterinary treatment costs, diagnostic testing, forage replacement, and additional management practices required to prevent further intoxication. Economic losses may also result from the need to dilute or discard high-nitrate forages, delayed grazing, and reduced forage utilization. Consequently, routine forage and water testing, gradual adaptation to nitrate-containing feeds, and proper nutritional management are critical strategies for minimizing production losses and improving the profitability of livestock operations [47].

## 15. Need for Additional Research

Despite ongoing efforts to understand and manage nitrate toxicity in cattle, several important knowledge gaps remain. Variations in feeding practices, forage preservation methods, and ruminal conditions all contribute to the complexity of nitrate management. Addressing these uncertainties through targeted research is essential for developing practical, science-based strategies that help producers minimize risk while maximizing forage utilization. The following sections highlight key areas where further investigation is needed.

### 15.1. Grazing Versus Conserved Forage Feeding

A more thorough understanding is needed of how nitrate metabolism varies between cattle grazing pasture and those consuming conserved forages such as silage or hay. Research indicates that grazing ruminants retain only 20–30% of the nitrogen they ingest in forage for productive purposes like milk or meat synthesis [48,49]. In less intensively managed grazing systems, animals often selectively consume leafy plant parts while avoiding stems, which tend to have higher nitrate concentrations. This selective behavior, combined with a more gradual exposure to nitrates, may enable rumen microbes to adapt and enhance their capacity to detoxify nitrate [50]. Gaining a better understanding of nitrate dynamics across both grazing and conserved feeding systems could help producers safely manage high-nitrate forages and reduce the risk of toxicity in cattle.

### 15.2. Fate of Nitrate in Forage Preservation Techniques

There is limited data on how different preservation methods, such as ensiling, haymaking, or dehydration, affect nitrate concentrations in stored forages. While some evidence suggests that fermentation during ensiling can lower nitrate levels, the degree of this reduction varies depending on the crop, moisture content, and microbial activity. More research is needed to measure nitrate changes during preservation and to develop best practices for safe storage and feeding.

### 15.3. Ruminal Environment and Feed Nitrate Breakdown

Multiple factors, including microbial population, ruminal pH, and the availability of electron donors, influence the efficiency of nitrate reduction in the rumen. However, many of these interactions remain poorly quantified, especially in extensive feeding situations. Further research is required to understand how diet composition, feeding rate, and animal health influence the fate of nitrate once ingested, particularly under subacute or chronic exposure scenarios.

## 16. Conclusions

The lack of ongoing research on nitrate toxicity has led to inconsistent feeding guidelines, creating uncertainty for cattle producers. This makes managing nitrate-rich forages particularly difficult during times of environmental stress or when relying on conserved feeds. A limited understanding of how ruminal conditions influence nitrate and nitrite reduction, as well as how cattle adapt to varying nitrate exposures, relate to the risk of toxicity. These knowledge gaps not only pose threats to animal health and performance but also have the potential to cause significant economic losses. Expanding research in this area is essential to establish clear, consistent, and practical guidelines that optimize forage use across the diverse feeding systems found in cattle production.

## Figures and Tables

**Table 1:** Variable maximum tolerable nitrate concentrations in feed for non-pregnant and pregnant cattle by state in the USA.

State	Non-Pregnant, mg/kg	Pregnant, mg/kg	Reference
Montana	10,000	5000	[4]
Nebraska	15,000	9300	[27]
Wyoming	17,720	15,505	[5]
North Dakota	17,720	15,505	[29]
South Dakota	17,720	15,505	[30]
Oklahoma	10,000	5000	[31]

## Data Availability

No new data were created or analyzed in this study. Data sharing is not applicable to this article.
